# Mesenchymal Stem Cell exosome delivered Zinc Finger Protein activation of cystic fibrosis transmembrane conductance regulator

**DOI:** 10.1002/jev2.12053

**Published:** 2021-01-23

**Authors:** Olga Villamizar, Shafagh A. Waters, Tristan Scott, Nicole Grepo, Adam Jaffe, Kevin V. Morris

**Affiliations:** ^1^ Center for Gene Therapy City of Hope–Beckman Research Institute at the City of Hope Duarte California USA; ^2^ Faculty of Medicine School of Women's & Children's Health University of New South Wales (UNSW) Sydney NSW Australia; ^3^ Molecular and Integrative Cystic Fibrosis Research Centre (miCF_RC) Faculty of Medicine University of New South Wales Sydney NSW Australia; ^4^ Department of Respiratory Medicine Sydney Children's Hospital Sydney NSW Australia; ^5^ School of Medical Science Griffith University, Gold Coast Campus 1 Parklands Dr Southport QLD Australia

**Keywords:** CFTR, exosome, cystic fibrosis, MSC, Zinc Finger Protein

## Abstract

Cystic fibrosis is a genetic disorder that results in a multi‐organ disease with progressive respiratory decline which leads to premature death. Mutations in the cystic fibrosis transmembrane conductance regulator (CFTR) gene disrupts the capacity of the protein to function as a channel, transporting chloride ions and bicarbonate across epithelial cell membranes. Small molecule treatments targeted at potentiating or correcting CFTR have shown clinical benefits, but are only effective for a small percentage of individuals with specific CFTR mutations. To overcome this limitation, we engineered stromal‐derived mesenchymal stem cells (MSC) and HEK293 cells to produce exosomes containing a novel CFTR Zinc Finger Protein fusion with transcriptional activation domains VP64, P65 and Rta to target the CFTR promoter (CFZF‐VPR) and activate transcription. Treatment with CFZF‐VPR results in robust activation of CFTR transcription in patient derived Human Bronchial Epithelial cells (HuBEC). We also find that CFZF‐VPR can be packaged into MSC and HEK293 cell exosomes and delivered to HuBEC cells to potently activate CFTR expression. Connexin 43 appeared to be required for functional release of CFZF‐VPR from exosomes. The observations presented here demonstrate that MSC derived exosomes can be used to deliver a packaged zinc finger activator to target cells and activate CFTR. The novel approach presented here offers a next‐generation genetic therapy that may one day prove effective in treating patients afflicted with Cystic fibrosis.

## 1 SIGNIFICANCE STATEMENT

Exosomes are important mediators that facilitate intercellular communication and transfer of genetic material to recipient cells. Here, we take advantage of this inherent property and engineer MSCs to produce exosomes packaged with a Zinc Finger Activator targeted to the CFTR gene promoter. We find that treatment of CF patient derived HuBEC with the CFZF‐VPR containing exosomes results in significant activation of CFTR. The novel approach presented here represents an effective way to deliver therapeutic proteins to patients afflicted with CF.

## 2 INTRODUCTION

Cystic fibrosis (CF) is an autosomal recessive genetic disease caused by mutations in the *CFTR* gene [Riordan et al., 1989). The CFTR protein is a c‐AMP activated chloride ion channel present in many organs including the lungs, pancreas, and reproductive glands (Sheppard & Welsh, 1999). Mutations in the *CFTR* gene result in a frameshift mutation in the mRNA coding sequence that impinges protein processing, channel gating, conductance function and ultimately results in the expression of a relatively non‐functional CFTR protein. The most prevalent CFTR mutation is caused by the deletion of a phenylalanine residue at position 508 (F508del) in the CFTR protein, which affects ∼70% of patients (Bobadilla et al., 2002). A small proportion of this misfolded F508del protein is however released from the Golgi and presented on the plasma membrane with a reduction in channel gating activity (Du et al., 2005, Kim & Skach, 2012, Thomas et al., 1992). There are a plethora of other CFTR mutations (Romey, 2006), which ultimately result in diminished CFTR protein function, and simply increasing its expression could improve the CF phenotype. A means to target and activate CFTR, irrespective of the variation in CFTR mutations, could prove therapeutically relevant in CF patients.

Novel targeted therapeutic approaches have emerged for the treatment of CF using small molecule modulators to enhance or restore the functional expression of mutated CFTR (Lopes‐Pacheco, 2016). CFTR modulators ivacaftor (Kalydeco®), lumacaftor/ivacaftor (Orkambi®), tezacaftor/ivacaftor (Symdeko®), and elexacaftor/tezacaftor/ivacaftor (Trikafta™) have been approved by the U.S. Food and Drug Administration for those people with specific CF mutations (Chaudary, 2018). Treatment with more than one CFTR modulator appears to be the most optimal strategy for many CFTR mutations, to improve CFTR function by enhancing cellular chaperone function VX‐809 (lumacaftor) and acting as potentiator that increases channel gating and conductance VX‐770 (ivacaftor) (Van Goor et al., 2011).

While this new line of therapeutics vastly improves the current standard of care for a subset of CF patients, it is only beneficial for those individuals with eligible CFTR mutations. A method that could result in a sustained increase in cell surface expression that is recalcitrant to the various mutant forms of CFTR could prove clinically impactful for those individuals who are refractory to the current modulator therapy. Towards this goal we developed a zinc finger activator protein targeted to the promoter for CFTR (CFZF‐VPR), as a means to transcriptionally activate CFTR expression.

The engineering of polydactyl zinc finger transcription factors has made it possible to recognize DNA sequences of 9–18 bp with high affinity (Mandell & Barbas, 2006). Similar artificial transcription factors have been designed to target regulatory regions of gamma globin gene promoters, resulting in the targeted increase of fetal hemoglobin (Graslund et al., 2005) and we recently developed a novel recombinant zinc finger protein transactivator, which specifically and potently targeted the activation of latent HIV‐1 (Scott et al., 2020). While promising, the enigma of how to deliver these synthetic transcription factors to cells has remained the rate limiting step in adapting synthetic zinc finger technologies as *bona fide* therapeutics.

One means to deliver zinc finger proteins (ZFP) intracellularly is by cell‐derived exosomes. The emerging field of extracellular particles and exosomes, collectively referred to here as exosomes, offers a unique, elegant opportunity to utilize basic cellular processes of exosome genesis to produce and deliver biologically relevant synthetic proteins. Exosomes are a subgroup of extracellular vesicles (EVs) originating in multivesicular bodies secreted into the extracellular environment after fusion with the plasma membrane (Fevrier & Raposo, 2004, Turturici et al., 2014). Exosomes contain mRNAs, non‐coding RNAs and proteins and transfer their contents from donor to recipient cells resulting in functional changes of target cells (Desrochers et al., 2016, Pegtel, 2019, Valadi et al., 2007). Thus, exosomes are important mediators that facilitate intercellular communication without direct cell‐to‐cell contact (Bang & Thum, 2012). Among exosomes derived from various cellular origins, mesenchymal stem cell‐derived exosomes have gained much attention for their potential to deliver diverse biomolecules (Phan et al., 2018) and modulate states of inflammation (Romanelli et al., 2019). Due to their inherent anti‐inflammatory properties (Romanelli et al., 2019), and observations that inflammation is observed in CF (Lin et al., 2018), we selected MSCs to produce engineered exosomes packaged with the CFTR activator CFZF‐VPR. We report here that MSC‐derived CFZF‐VPR containing exosomes can functionally activate CFTR expression in both HEK293 cell lines and HuBECs. Collectively, the observations presented here demonstrate that exosomes carrying the transcriptional activator CFZF‐VPR can target and functionally activate CFTR.

## 3 RESULTS

### 3.1 Enhanced CFTR expression in HuBECs by the CFZF‐VPR

**FIGURE 1:**
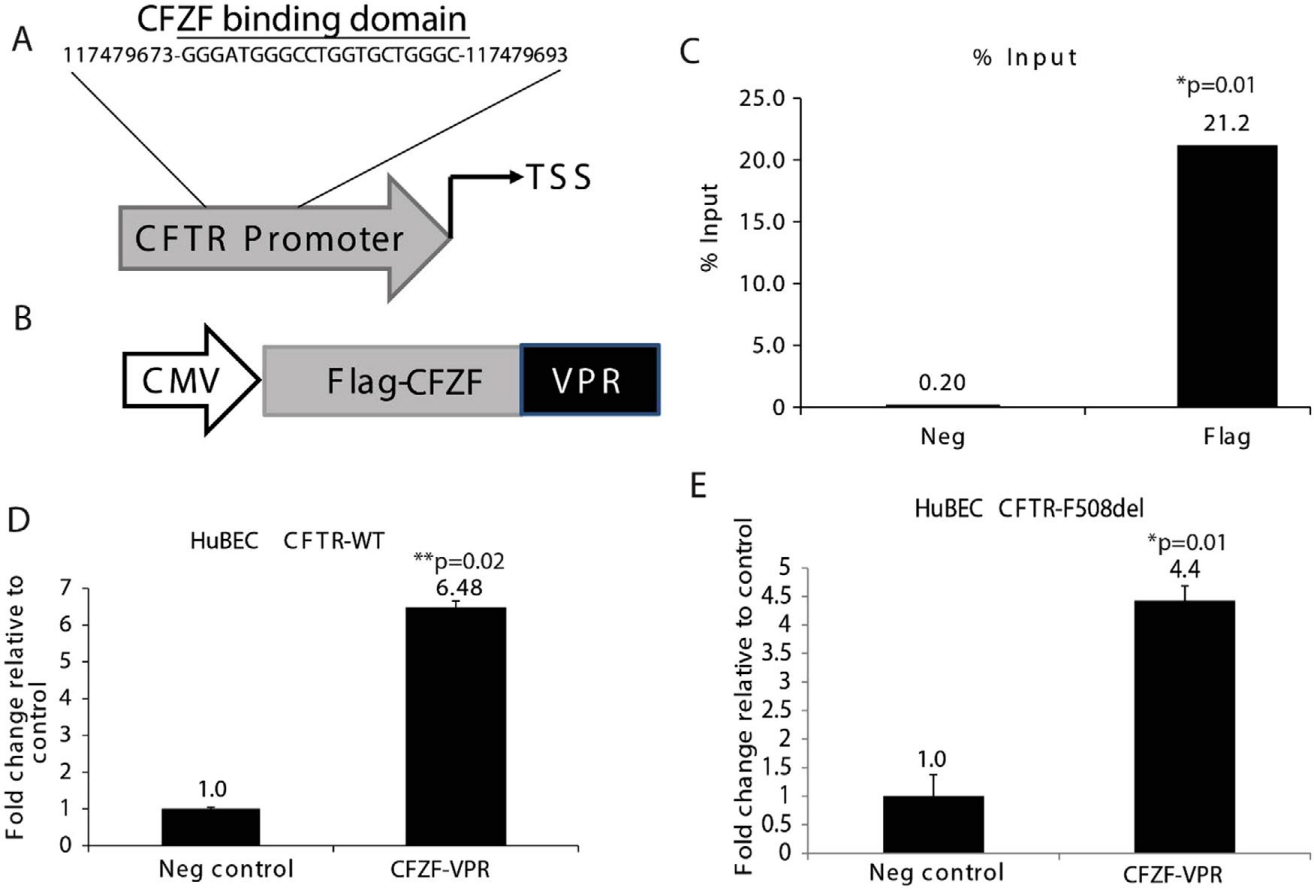
CFTR gene activation using Zinc Finger activator protein. (A) A schematic of the zinc finger binding domain targeted by CFZF in the CFTR promoter. The target sequence is shown along with its genomic location as determined from the UCSC genome browser. (B) Schematic of vector expressing the FLAG‐CFZF‐VPR. A CMV Pol II promoter expresses a FLAG‐tagged CFZF targeted to the CFTR promoter with a VPR activation domain. (C) ChIP assays were performed and enrichment relative to control (Neg) is shown as a fraction of input. (D) CFTR mRNA levels as determined by qRT‐PCR in HuBECs from healthy donor (HuBEC CFTR‐WT), and (E) HuBECs from CF patient with the F508del mutation (HuBEC CFTR‐F508del). HuBEC non‐transfected were used as negative control (Neg control). Experiments show the standard error of the mean and p values are represented (paired two‐sided Student's T‐test, *P = 0.01, **P < 0.02)

We investigated a synthetic zinc finger protein fused to VP64 (Herpesvirus transcription factor), p65 (NFкB subunit), and Rta (an activator of Epstein‐Barr virus genes)(VPR) (Mandell & Barbas, 2006) to specifically activate the CFTR gene. The zinc finger protein (CFZF), targeted to the CFTR promoter, was designed using the zinc finger tools software™ and fused to VPR (Scott et al., 2020) to generate CFZF‐VPR (Figure 1A, Table S1). To test whether CFZF‐VPR binds directly to the CFTR promoter, a FLAG‐tag ZFP‐VPR vector was constructed with a CMV promoter used to express the transgene (Figure 1B), and used in a Chromatin Immunoprecipitation assay (ChIP) (Brena et al., 2006). ChIP analysis verified that the CFZF‐VPR is enriched at the CFTR promoter relative to the non‐transfected HuBEC used as negative controls (Figure 1C). Next, we tested whether the CFZF‐VPR was able to activate mutant CFTR transcription. Both wild‐type (WT) and the F508del‐CFTR HuBECs were found to exhibit significant transcriptional activation of CFTR following transfection with CFZF‐VPR (Figures 1D,E), suggesting that CFZF‐VPR is a transcriptional activator of CFTR which can activate both WT and F508del‐CFTR variants. Collectively, these data demonstrate the CFZF‐VPR binds the CFTR promoter and activates transcription.

### 3.2 Mesenchymal Stem Cell CFZP‐VPR packaged exosomes activate CFTR expression in target cells

**FIGURE 2:**
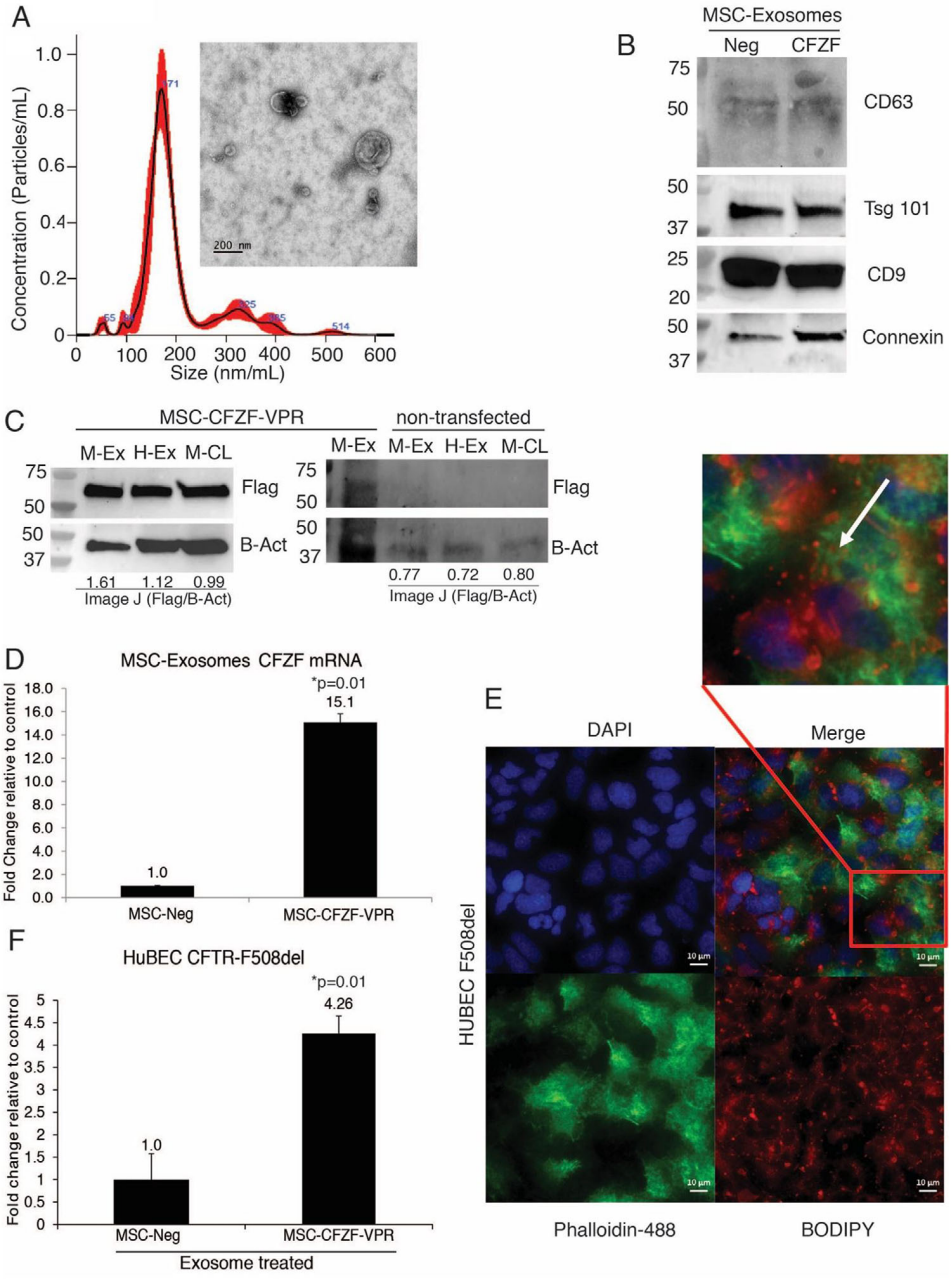
MSC Exosome mediated delivery of Zinc Finger Protein Activator increases the expression of CFTR. (A) TEM micrograph of exosomes isolated from the culture medium of MSC‐CFZF‐VPR transfected cells. Exosomes were measured by using Nanosight NS 300 system in the supernatant from cultures cells. The histogram represents particle size distribution. (B) Western blot analysis for exosome markers in CFZF‐VPR transfected MSC (CFZF) and non‐transfected MSCs (Neg) derived exosomes. (C) Western blot of FLAG‐tagged CFZF‐VPR protein enriched in exosomes from left: MSC‐CFZF‐VPR or right: non‐transfected MSC‐exosomes (M‐Ex), HEK293‐exosomes (H‐Ex) and corresponding MSC cell lysate (M‐CL) samples, respectively. The Image J values from the Flag‐tag (Flag) relative to Beta Actin (B‐Act) are shown below the blot. (D) Evaluation by qRT‐PCR of mRNA expression of exosomes from CFZF‐VPR/Cx43‐transfected MSCs. The results from triplicate exosomes collected from three different CFZF‐VPR/Cx43 transfected MSCs are shown. (E) Light microscopy immunofluorescence images of HuBECs uptake of MSCs‐CFZF‐VPR exosomes labelled with BODIPY TR ceramide (red), Nuclei (Blue), Actin (Green). Scale bar, 10 μm. (F) CFTR expression was determined by qRT‐PCR following treatment with MSC exosomes directed to the CFTR promoter (MSC‐CFZF‐VPR) or Control (MSC‐Neg) in HuBECs. For E and F experiments were performed in triplicate with 10e+03 HuBECs treated with 5e+10 exosomes. Experiment shown the standard error of the means and p values from a paired two‐sided T‐test, *P = 0.01

Exosomes are promising tools for drug delivery (O'Brien et al., 2020). Here, we developed exosomes carrying CFZF‐VPR using a cell producer system by co‐transfecting plasmids encoding the CFZP‐VPR and Connexin 43 (Cx43), to facilitate endosomal release of payloads in target cells (Kojima et al., 2018). Exosomes were produced and isolated from both CFZF‐VPR/Cx43 transfected MSCs and HEK293 cells cultured in exosome‐depleted FBS medium and from non‐transfected cells which served as the negative control. The purified MSC‐CFZF‐VPR (Figure 2A) and HEK293 CFZF‐VPR exosomes (Figure S1A) were characterized for their size and form using nanoparticle tracking analysis (NTA) and TEM analysis with the majority of particles ranging between ∼150–170 nm in size. Importantly, exosomes from MSC or HEK293‐cells transfected with Cx43 had an increased amount of protein detected as well as several other known extracellular vesicles markers (CD63, CD9, and TSG101) (Figure 2B, Figure S1B top blot).

Furthermore, FLAG‐tagged CFZF‐VPR protein was observed in both MSC‐CFZF‐VPR and HEK‐CFZF‐VPR exosomes and from MSC lysate (M‐CL), but not in non‐transfected control samples (Figure 2C, Figure S1B top blot). CFZF‐VPR mRNA was also found to be packaged in both MSC and HEK293 derived exosomes (Figure 2D and Figure S1C, respectively). Additionally, we demonstrated exosome uptake by adding Bodipy‐labelled MSC‐derived exosomes into the F508del‐CFTR‐HuBEC cells (Figure 2E) and the CFZF‐VPR protein was detected in the recipient HuBECs treated with MSC‐CFZF‐VPR compared with non‐exosome treated (neg) HuBEC cells (Figure S1B bottom blot).

Next, we assessed the capacity of CFZF‐VPR containing exosomes to activate CFTR expression. We observed a significant increase in CFTR mRNA expression 48 h after treatment with either MSC or HEK293 derived CFZF‐VPR exosomes in HuBECs with the F508del‐CFTR mutation (Figure 2F and Figure S1E, respectively) and in WT‐CFTR HuBECs treated with MSC‐CFZF‐VPR (Figure S1F). These observations demonstrate that CFZF‐VPR delivered by exosomes can activate CFTR expression.

### 3.3 MSC‐CFZF‐VPR enhances CFTR protein expression and functionally improves chloride transport

**FIGURE 3:**
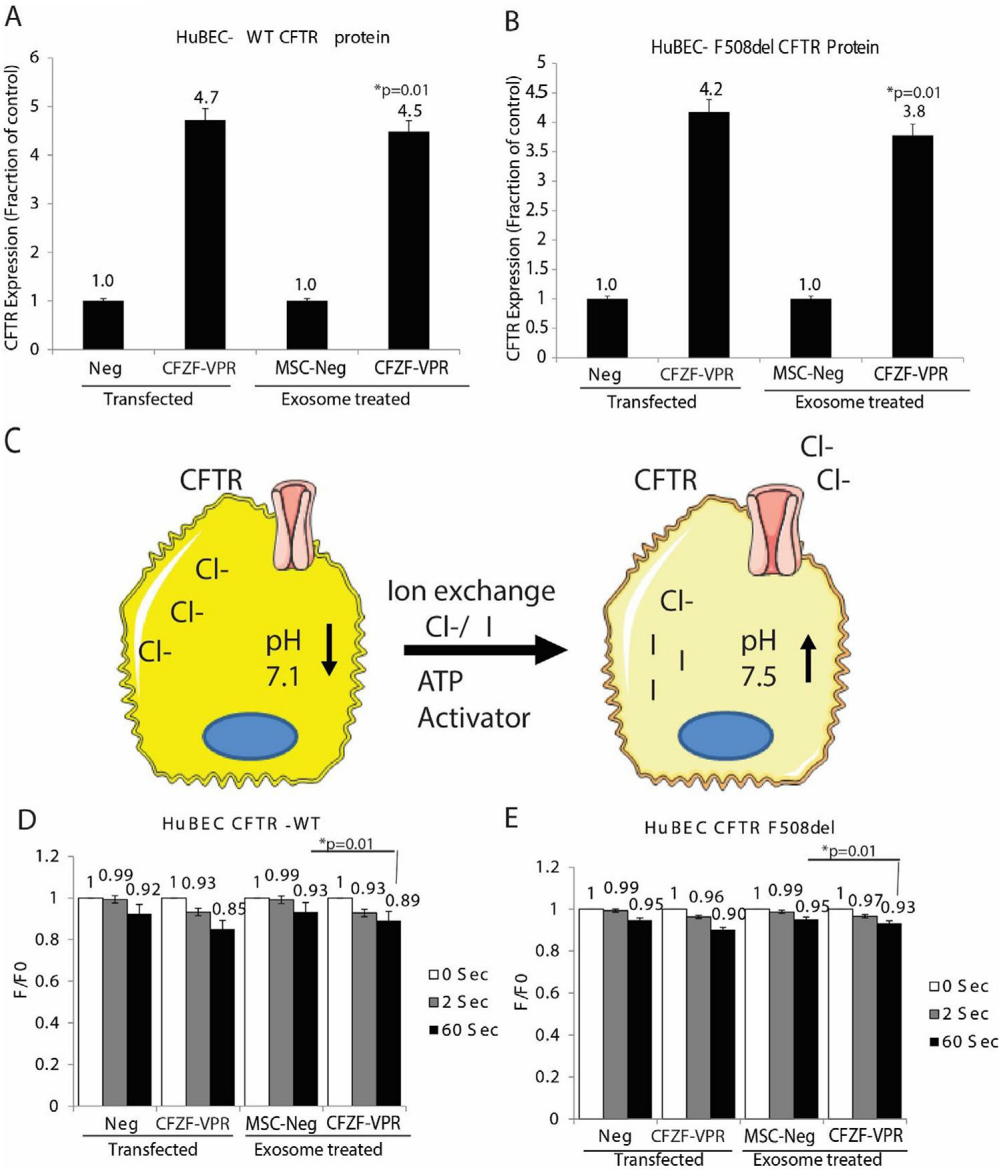
Exosome mediated delivery of CFZF‐VPR increases CFTR protein expression and enhanced Chloride transport. CFTR ELISA analysis of CFTR protein expression in (A) HuBECs from healthy donor HuBEC–WT and (B) in patient cells with F508del mutation following transfection with CFZF‐VPR plasmid or treated with MSC‐derived exosomes carrying CFZF‐VPR. (C) A schematic is shown depicting the halide assay used to assess chloride transport. Fluorescence decrease was evaluated at 0, 2, and 60 s in response to exchange of 25 mM of sodium iodide in cells treated with 0.3 μM of forskolin and combination of VX‐809 and VX‐770, current drugs used for treatment of CF patients. (D) HuBEC CFTR‐WT and (E) HuBEC F508del cells were transfected with CFZF‐VPR plasmid or treated with MSC‐derived exosomes carrying CFZF‐VPR and assessed 48 h post‐treatment using the halide assay. Experiments were performed in triplicate with 10e+03 HuBEC treated with 5e+10 exosomes. ELISA and halide experiments were performed in triplicate in cells shown with the standard error of the means and p values from a paired two‐sided T‐test, *P < 0.05

In order to investigate the effect of the MSC‐derived exosomes containing CFZF‐VPR in modulating the expression of CFTR gene we evaluated the CFTR protein expression in differentially treated cells. We found that treatment of both HuBEC‐WT and HuBEC‐F508del cells with the CFZF‐VPR containing exosomes resulted in a significant increase in CFTR protein compared to those cells treated with control exosomes, and at equivalent levels of activation to plasmid transfection (Figures 3A,B). These data demonstrate that MSC derived CFZF‐VPR containing exosomes can induce F508del‐CFTR protein expression. Notably, we also detected an increase in the CFTR protein in HuBEC‐F508del cells treated with HEK293 derived exosomes carrying the CFZF‐VPR (Figure S2A), suggesting that the observed exosome‐mediated activation of CFTR is independent of the parent producer cells.

To determine if the observed increased CFTR protein expression was functionally relevant, we measured CFTR membrane activity using a halide assay which evaluates the increase of ion transport within cells expressing iodide‐sensitive yellow fluorescent protein (YFP) (Singh et al., 2020), by using forskolin stimulation of CFTR and replacing chloride ions with iodide in cells expressing mutated YFP (Figure 3C). To assess if CFZF‐VPR containing exosomes functionally corrected the CFTR anion channel defect in vitro we evaluated the changes in YFP fluorescence in HuBECs pre‐incubated with CFTR activator forskolin, treated with MSC‐CFZF‐VPR or controls in the presence of the potentiator (VX770) and the corrector (VX809) (Janas et al., 2015, Janas et al., 2015). In both, wild‐type and F508del HuBECs transfected with CFZF‐VPR plasmid or treated with MSC exosomes containing CFZF‐VPR, we observed a significant decrease in fluorescence when compared to those cells treated with control exosomes (Figures 3D,E, respectively). A reduction of fluorescence was also observed in HuBEC F508del treated with HEK293 exosomes containing CFZF‐VPR (Figure S2B). These observations demonstrate that MSC and HEK293 engineered exosomes can functionally deliver a CFZF‐VPR to primary CFTR‐F508del bronchial epithelial cells to correct the anion channel defect in vitro.

## 4 DISCUSSION

Zinc finger protein activators are a promising therapeutic tool for clinical applications to activate endogenous gene expression (Mandell & Barbas, 2006, Scott et al., 2020). Several studies have explored the potential of this approach to modulate the expression of genes such as gamma globin to increase expression of fetal hemoglobin in human erythroblast (Wilber et al., 2010) or for the targeted activation of latent HIV reservoirs in primary human CD4+ T‐cells (Perdigao et al., 2020, Scott et al., 2020). Herein, we designed a zinc finger protein activator, CFZF‐VPR, targeting the CFTR promoter and demonstrated the successful induction of robust CFTR expression in primary human bronchial epithelial cells with the F508del mutation. These observations suggest CFZF‐VPR induced endogenous CFTR gene expression may prove useful as a means to treating CF and that this approach is largely independent of the dominant F508del mutation found in the CFTR gene.

Exosomes have proven to be stable, biocompatible modalities to deliver a specific protein, miRNA or drug (Popowski et al., 2020) and represents a valuable tool to deliver therapies to tissue in otherwise difficult to treat diseases such as CF. Our previous study demonstrated the exosome mediated delivery of modified antisense oligonucleotides into nasal epithelial cells with the F508del mutation, which corrected the CFTR function (Villamizar et al., 2019). Other groups have also demonstrated exosome delivery of siRNA and microvesicle‐mediated delivery of CFTR protein into differentiated human airway epithelial cells providing a powerful genetic tool to functionally correct the Cl‐ channel defect in vitro (Singh et al., 2020). Notably, MSC derived exosomes have been used as a treatment for diseases and disorders of many systems such as the cardiovascular, neurological, musculoskeletal and immune system (Phan et al., 2018), suggesting that they are clinically safe and efficacious. Recent studies have also demonstrated that using exosomes alone from engineered MSCs overexpressing the SARS CoV‐2 protein receptor angiotensin‐converting enzyme 2 (ACE2) efficiently inhibited virus infection and reduced lung injury (Inal, 2020), suggesting that MSC exosomes can localize to lung. Building on these observations we engineered MSCs to become CFZF‐VPR exosome packaging machines that can transmit not only proteins, mRNA and non‐coding RNAs to mediate cell‐to‐cell communication and regulate recipient cell function (Liu & Su, 2019), but also to deliver a CFTR promoter specific CFZF‐VPR mRNA and protein. We find that these exosomes delivered functional CFZF‐VPR which rescued CFTR function in HuBECs with F508del mutation by increasing the levels of CFTR protein expression and function.

The incorporation of the CFZF‐VPR mRNAs into the EVs from the parent cells can occur based on the lipid‐mediated RNA loading mechanism. Previous studies have proposed that RNA is loaded into exosomes when it binds to the raft‐like region during the budding process (Janas et al., 2015). RNA is transported by RNA‐binding proteins (RBPs), interacting with the cytoplasmic surface of the multi vesicular bodies (MVB) limiting membrane. RNAs with the highest affinity to the raft‐like region are retained at the membrane. The affinity of an RNA molecule to the raft‐like region is thought to be determined by binding motifs in the RNA sequence as well as by hydrophobic modifications to the RNA (Janas et al., 2015).

**FIGURE 4:**
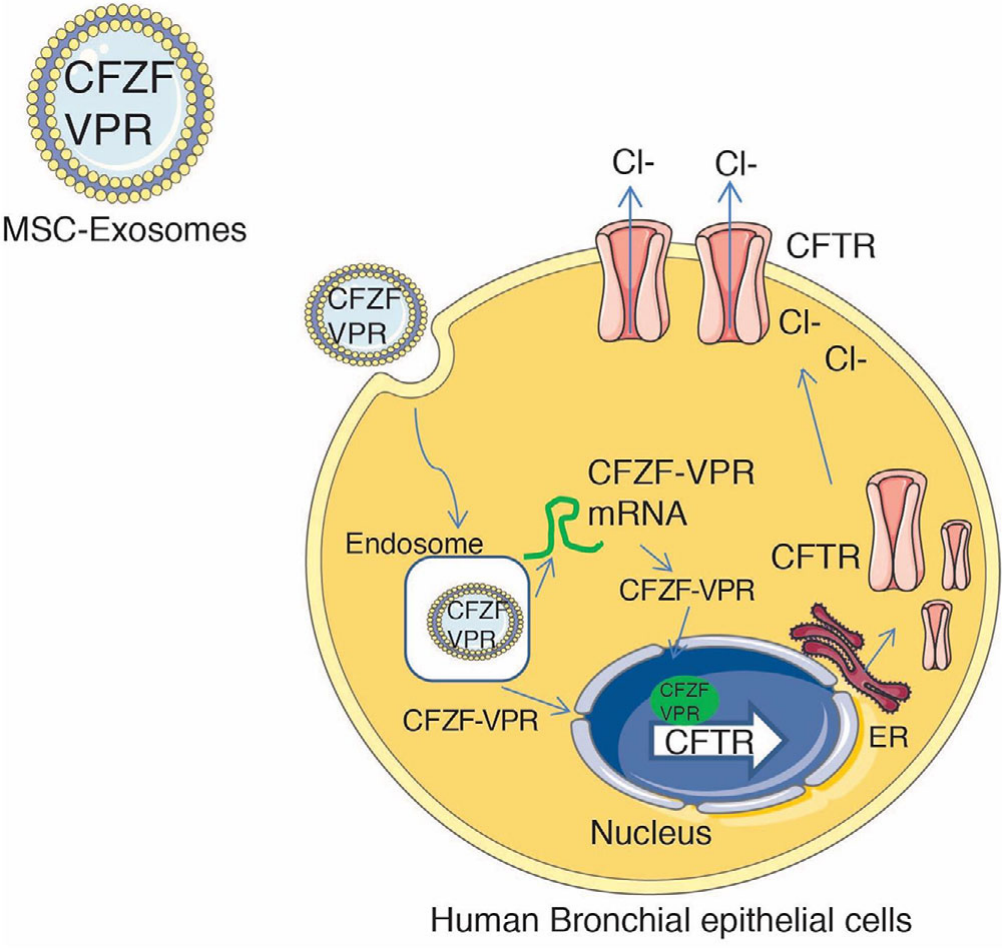
Underlying mechanism of MSC‐CFZF‐VRP exosome mediated activation CFTR. MSC‐exosome are generated to contain a CFZF‐VPR targeted to the CFTR promoter and are internalized into human bronchial epithelial cells whereby the CFZF‐VPR protein and CFZF‐VPR mRNA are released into the intracellular environment by the action of Cx43 and presumably interactions with other endogenous exosome pathway proteins. The result of this Cx43 mediated delivery of CFZF‐VPR is increased expression and functional activation of CFTR

We also find here that the overexpression of Cx43 in the MSC and HEK293 exosome producer cells was found to significantly accentuate transfer of information, both CFZF‐VPR RNA and protein from exosomes to target cells. This observation is noteworthy as it suggests that mutant Cx43 (S368A) over‐expression is a useful delivery helper and collectively with CFZF‐VPR proves to be a novel means to deliver functional therapeutic zinc finger activators to treat CF patients (Figure 4). The presence of Cx43, a gap junction protein that links adjacent cells, on the exosome membrane surface facilitates the direct delivery of exosome cargo to recipient cells by interacting and facilitating the fusion of EV surface proteins with the target cell membrane (Gemel et al., 2019, Soares et al., 2015). Functionally, the mutant Cx43 incorporation is expected to inhibit serine phosphorylation, an action that is presumed to facilitate the formation of a hemichannel that might accentuate the delivery of proteins into the recipient cells (Martinovich et al., 2017)(Figure 4). Supporting this notion, we find using endocytosis inhibitor Genistein that there is an inhibition of the MSC derived CFZF‐VPR exosome uptake in HuBECs (Figure S3). These data suggest that MSC‐derived exosomes loaded with CFZF‐VPR might be internalized in the cells through a clathrin‐independent endocytic mechanism. However, to what extent this molecular pathway was engaged in the observations presented here remains to be determined.

Collectively, the observations presented here represent, to our knowledge, the first utilization of engineered MSC exosomes as a method to deliver both zinc finger activator proteins and mRNAs to increase CFTR expression and might suggest that such a method could be expanded upon as a genetic therapy to therapeutically bolster CFTR expression in those patients that do not respond to the current therapies or as an adjunctive cellular therapeutic approach to treating CF.

## 5 MATERIALS AND METHODS

### 5.1 Cell culture

The Human basal bronchial epithelial cells (HuBEC) from healthy volunteers or CF patients were obtained from bronchoalveolar lavage fluid (BALF) of donors during bronchoscopy. The participants’ provided written informed consent. The sample collection was approved by the Sydney Children's Hospital Ethics Review Board (HREC/16/SCHN/120). Basal bronchial cells were conditionally reprogrammed using F‐medium, Rock inhibitor and irradiated NIH3T3s feeders based on a previously published protocol (Martinovich et al., 2017). At confluency, cells were dissociated using a differential trypsin method and cryopreserved. The HuBEC WT genotype: CFTR wt/wt (normal)), HuBEC CF patients (genotype: CFTR F508del/F508del (CF)) were maintained in Pneumocult™ EX Plus medium (Stemcell Technologies) at 37°C and 5% CO_2_.

### 5.2 Exosomes Isolation and characterization

Human bone marrow‐derived mesenchymal stem cells (hBM‐MSCs) were obtained from STEMCELL Technologies™. BM‐MSCs were expanded in serum‐free, conditions (MesenCult®‐ACF‐XF Attachment Substrate, STEMCELL Technologies) according to the manufacturer's instructions. HEK 293 cells were cultured in Dulbecco Minimum Essential Medium (DMEM) (Mediatech, Manassas, VA) supplemented with exosome depleted serum (Gibco,USA), 50 μg/ml Pen/Strep (Mediatech, Houston, Texas, USA) at 37°C and 5% CO_2_. Six million cells were co‐transfected with 15 μg of pCDNA3.1‐CFZF‐VPR and 5 μg of pDB68 encoding Cx43 S368A, a constitutively active mutant of Cx43 (PhCMV Cx43S368A‐pA) (Kojima et al., 2018), plasmids using VIROMER®. The resultant supernatant was collected after 48 h from HEK293 cells and 96 h for MSCs and centrifuged at 800 × *g* for 5 min, followed for an additional spin at 2000 × *g* for 10 min, supernatant was filtered (0.2 μm) and the ultracentrifuge 100,000 × *g* for 2 h at 4°C. Pelleted exosomes were re‐suspended in PBS and storage at −80°C.

### 5.3 Western Blot

Detection of exosomal protein markers was performed using Western Blot. Exosomes and cells were lysed using RIPA buffer (ThermoFisher, USA). 5e+13 particles of extracellular vesicles (EVs) (80–100 μg protein) or/and whole‐cell protein (20–50 μg) extract were boiled in 4X laemmli sample buffer (Bio‐rad, USA) and loaded onto 4–15% Mini‐PROTEAN TGX™ gels (Bio‐Rad, USA). Following electrophoresis (100V, 30 mA), the proteins were transferred to a PVDF membrane. The membranes were blocked with 5% non‐fat dry milk in TBST and then incubated overnight with primary antibodies CD9 (sc‐13118, Santa Cruz, USA), TSG101 (sc‐136111, Santa Cruz, USA), CD63 (sc‐5275, Santa cruz, USA), Connexin 43 (3D8A5, Invitrogen, USA), Beta Actin (MA1‐140, Invitrogen, USA), Anti‐Flag M2 (F3165, Sigma, USA). After washing, the membrane was incubated with horseradish peroxidase–conjugated Rabbit anti‐mouse IgG and then subjected to enhanced chemiluminescence using super signal West Pico HRP substrate (Thermo Scientific, USA).

### 5.4 Identification of particles by nanoparticle tracking analysis

Nanoparticle tracking analysis (NTA) measurements were performed by using a NanoSight NS300 instrument. The capture settings were Camera Type: sCMOS, Laser Type: Blue488, Camera Level: 9, Slider Shutter: 607, Slider Gain: 15, FPS 25.0, Number of Frames: 1498, Temperature: 23.1‐23.1°C, Dilution factor: 1000 and Syringe Pump Speed: 30. The size distribution and quantification of exosome preparations were analysed by measuring the rate of Brownian motion with a NanoSight LM10 system (NanoSight, Wiltshire United Kingdom) equipped with fast video capture and particle‐tracking software NTA 3.3 ‐ Sample Assistant Dev Build 3.3.302. Isolated exosomes were diluted (1:1000) and injected into a NanoSight sample cubicle. The mean ± SD size distribution of exosomes was determined as well as the mean number of particles per milliliter. For mesenchymal stem cell derived exosomes concentration was: 5.94e+11 particles/ml.

### 5.5 Negative staining electron microscopy of extracellular vesicles

Specimens at certain concentrations were adsorbed to glow‐discharged, carbon‐coated 200 meshEM copper grids. Samples were prepared by conventional negative staining with 1% (w/v) uranyl acetate. EM images were collected with an FEI Tecnai 12 transmission electron microscope (Thermo Fisher Scientific, Waltham, MA, USA) equipped with a LaB6 filament and operated at an acceleration voltage of 120 kV. Images were recorded with a Gatan 2× 2 k CCD camera (Gatan, Inc., Pleasanton, CA, USA) at a magnification of 100 μm and a defocus value of ∼1.5 μm.

### 5.6 Exosome labelling

Exosomal membrane was labelled with BODIPY TR ceramide, according to the manufacturer's protocol (Molecular Probes/Invitrogen Life Technologies, Carlsbad, California, USA). Briefly, 5e+10 exosomes were stained with 10 μmol/l BODIPY TR ceramide. Excess fluorescent dye was removed by using Exosome Spin Columns (Life Technologies, Carlsbad, California, USA). BODIPY‐labelled exosomes were dropped on 10e+3 cells and incubated in exosome depleted medium at 37°C and 5% CO_2_. Bodipy‐labelled exosomes uptake was evaluated by light microscopy.

### 5.7 Construction of CFZP‐VPR expression plasmids

The cloning was performed as described before (Scott et al., 2020). Briefly, a Zinc Finger directed to the CFTR promoter was designed using the zinc finger tools software™, ordered as a gBlock (IDT, USA) and sub‐cloned into the pcDNA3.1 plasmid encoding a ZFP‐362‐ VPR using the NEBuilder® HiFi DNA assembly method to replace the ZFP‐362 with ZFP directed to CFTR promoter (*CFZF‐VPR)*. HuBEC were transfected with 2 μg of CFZF‐VPR plasmid using VIROMER® Red according to the manufacturer's instructions and cultured for 48 h.

### 5.8 qRT‐PCR analysis of gene expression

To determine transcript levels of CFTR total RNA was isolated 48 h post‐transfection using the Maxwell 16 LEV simplyRNA purification kit and the Maxwell 16 Research Instrument (Promega, Madison, WI). DNase‐treated RNA samples were then standardized and reverse transcribed with QuantiTect (Qiagen) using an oligo‐dT/random monamer primer mix. Quantitative real‐time polymerase chain reaction (qRT‐PCR) was carried out using Kapa Sybr Fast universal qPCR mix (Kapa Biosystems, Wilmington, MA) on an Eppendorf Mastercycler realplex. Thermal cycling parameters started with 3 min at 95°C, followed by 40 cycles of 95°C for 3 s and 60°C for 30 s. The fold change in gene expression was calculated using the 2^–ΔΔCt^ method. Following the RT step, qRT‐PCR was carried out with CFTR and β‐Actin primers (**Table S1**).

### 5.9 Chromatin immunoprecipitation assay (Brena et al., 2006)

ChIP was performed as described before (Ray et al., 2019). Briefly, using 5 × 10^6^ A549 cells transfected with 5 μg of pCDNA‐CFZP‐VPR, after 48 h cells were cross‐linked, and nuclei was digested using microccocal nuclease. 10% of the nuclei was set aside for input and the rest was incubated in 10 μg of anti‐FLAG antibody overnight at 4°C. Thereafter, 50 μl of magnetic protein A/G beads were added and incubated for 2 h. Samples were eluted reverse crosslinked at 65°C and treated with RNase and proteinase K. Then, DNA was isolated (ZYMO, USA) and used to perform qPCR. The calculated concentrations were used to determine the enrichment of the protein at the target DNA site, as a fraction of input.

### 5.10 Halide assay

Stable cells expressing EYFP were obtained by transducing CFPAC with pLenti‐EYFP plasmid and single cell sorted to select an EYFP cell clone. Human nasal cells WT and F508del expressing transiently EYFP by delivery of Premo™ Halide Sensor (Thermo Fisher Scientific, Waltham, MA, USA) by BacMam technology ‐ Pharmacologically relevant—known modulators show dose‐dependent quenching and BacMam delivery enables assays in primary cells. The assay combines the YFP Venus halide sensor with a surrogate ion for chloride (iodide); upon stimulation of the chloride channel or transporter, iodide ions flow down the concentration gradient into the cells and quench YFP fluorescence upon binding; the amount of quench is directly proportional to the ion flux (chloride channel or transporter activity). Cells cultured on 96‐well plates were treated with Forskolin, VX809, VX770, amilirate and niflumic acid assayed after using Halide stimulus buffer (NaI 25 mM) and fluorescence evaluated in a plate reader (GloMax®‐Promega, Madison, WI, USA). Normalization for expression levels was performed by baseline correction (F/F0).

### 5.11 Light microscopy and imaging

Cells were cultivated fixed with BD cytofix/cytoperm™ fixation/permeabilization Kit (Cat. No. 554715 BD bioscience, Franklin Lakes, NJ, USA) at room temperature for 20 min and then stained with fluorescent 488‐Phalloidin (green) for actin using the manufacturer's recommendations (Molecular Probes/Invitrogen Life Technologies). Fluorescence images were collected on Zeiss LSM‐700 confocal microscope. For the analysis of the cellular internalization of exosomes Zeiss acquisition parameters, including exposure, focus, illumination, and Z stack projection, were controlled by Zen 2012 Imaging Software for Acquisition and Analysis.

### 5.12 Enzyme‐linked Immunosorbent Assay (Nesterova et al., 2001)

Quantitative detection of human CFTR (Novus Biologicals Centennial, Co, USA) was performed according to the manufacturer's. Briefly, this sandwich ELISA utilizes a microplate coated with an antibody specific to human CFTR. Standards (100 μl) were added in duplicate to the plate. Nasal WT and F508del differentially treated cells were freeze‐thaw lysed and samples lysates (100 μl) were added in triplicate to the plate. Next, the plate incubated for 90 min at 37°C in the dark. After incubation the liquid was removed and 100 μl of biotinylated detection antibody working solution added to each well. The plate was covered with the sealer, gently mixed and incubated for 1 h at 37°C. The solution was aspired from each well and 350 μl of wash buffer added to each well and the plate incubated for 2 min at room temperature. Next the solution aspirated from each well followed by 3 washes after which 100 μl of HRP conjugate working solution was added to each well. The HRP exposed plate as was then covered with the plate sealer and incubated for 30 min at 37°C after which the solution was aspirated from each well and the wash step repeated five times after which 90 μl of substrate reagent was added to each well; the plate was covered with a new plate sealer and incubated for 15 min at 37°C in the dark followed by the addition of 50 μl of stop solution added to each well. The optical density (OD value) was determined for each well with the micro‐plate reader set to 450 nm. Specification of the kit: Sensitivity: 0.10 ng/ml, Detection Range: 0.16–10 ng/ml.

### 5.13 Statistical analysis

The results are presented as mean of standard error (SEM). For comparison of two groups, the unpaired Student's t‐test was used as analysed in Excel. Differences were considered statistically significant when *P* < 0.05.

## CONFLICT OF INTEREST

K.V.M and O.V. have submitted a provisional patent on this technology.

## Supporting information

Supporting Information
