# Supplementary material for: Mesenchymal Stem Cell exosome delivered Zinc Finger Protein activation of cystic fibrosis transmembrane conductance regulator
Source: J Extracell Vesicles. 2021 Jan 23;10(3):e12053. doi: 10.1002/jev2.12053 (PMC7825549; doi:10.1002/jev2.12053)
Supplement: Supplementary file 1 — Supporting Information [file JEV2-10-e12053-s001.docx]

**Supplemental Figures and tables**


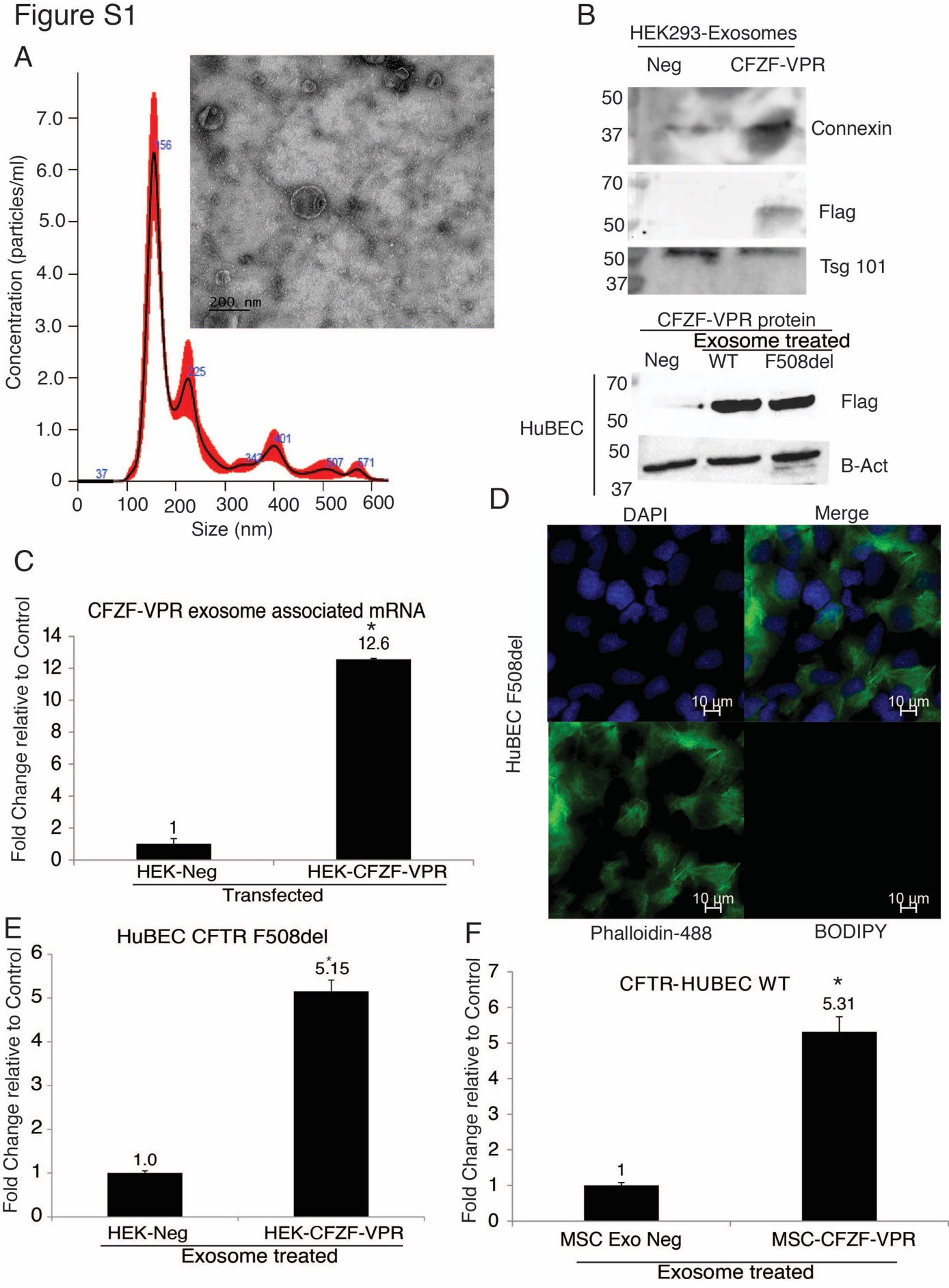


**Figure S1. HEK293 Exosome mediated delivery of CFZF-VPR increases expression of CFTR.** **(A)** TEM micrographs of CFZF-VPR containing exosomes isolated from the culture medium of HEK293 cells. Exosomes were measured by using Nanosight NS 300 system in the supernatant from cultures cells. The histogram represents particle size distribution. **(B)** Western blot analysis Top: for exosome markers in HEK293-derived exosomes from non-transfected HEK293 cells (Neg) and HEK293 cells transfected with (CFZF-VPR). Flag-tagged CFZF-VPR protein enriched in exosomes from CFZF-transfected HEK293. HuBEC cells are also shown Bottom: CFZF-VPR-flag tagged protein from WT HuBEC non-exosome treated (Neg) cells and WT and F508del HuBECs treated with MSC-derived exosomes loaded with CFZF-VPR. (**C**) CFZF-VPR expression in HEK293 derived exosomes as determined by qRT-PCR from CFZF-VPR/Cx43 transfected (HEK-CFZF-VPR) and control (293-Neg) HEK293 cells. **(D)** Light microscopy immunofluorescence images of HuBECs as negative control (no-exosomes) for uptake with BODIPY TR ceramide (red), Nuclei (Blue) Actin (Green), Scale bar, 10 μm. **(E)** CFTR transcript expression after treatment with HEK293-derived exosomes directed to the CFTR promoter (HEK-CFZF-VPR), Control (293-Neg) in F508del and **(F)** CFTR transcript expression after treatment with MSC-derived exosomes directed to the CFTR promoter (MSC-CFZF-VPR) in CFTR-HuBEC WT cells. Experiments were performed in triplicate with 10e+03 HuBEC treated with 5e+10 exosomes. Experiment shown the standard error of the means and p values from a paired two-sided T-test, *p= 0.01.


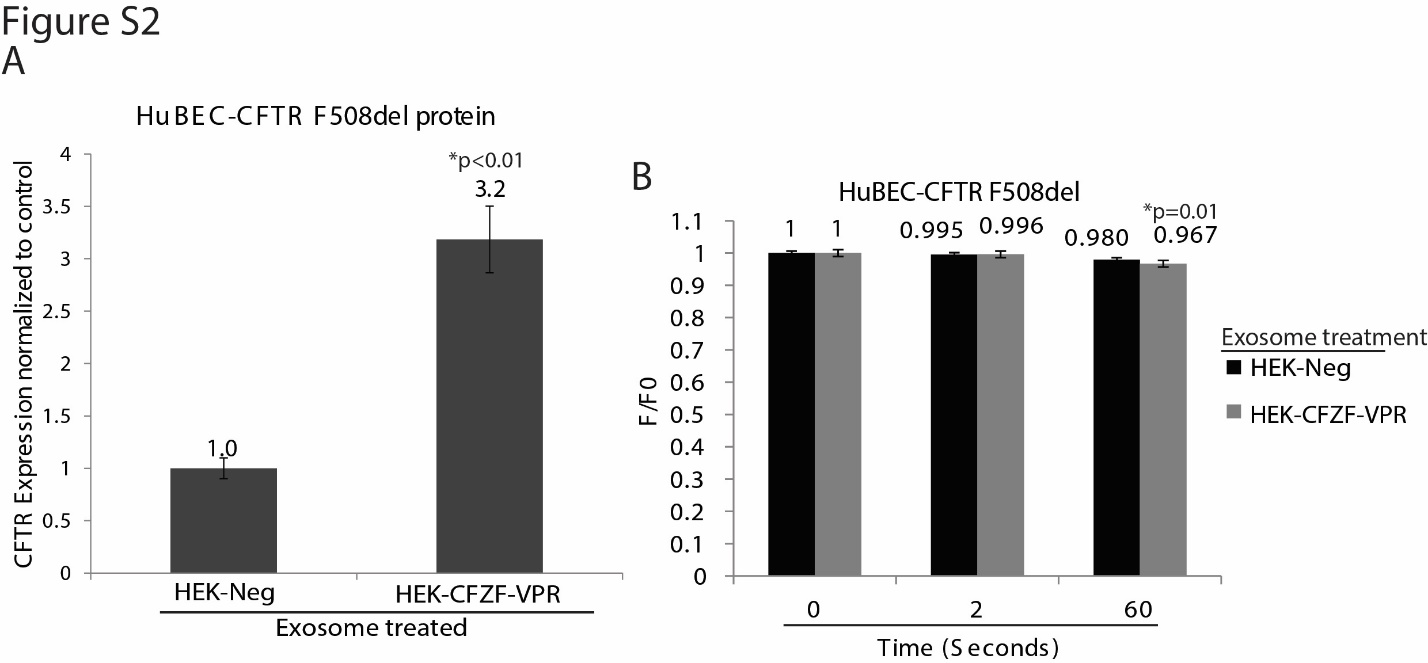


**Figure S2.** **HEK293 exosome mediated delivery of CFZF-VPR enhances CFTR Chloride transport. (A)** CFTR ELISA showing CFTR protein levels expression increased in HuBECs from CF patients with F508del mutation after treatment with HEK293 CFZF-VPR packaged exosomes (HEK-CFZF-VPR) relative to HEK293 cell control exosome treatment (HEK-Neg). **(B)** CFTR-mediated Halide transport in EYFP showed fluorescence decrease in F508del HUBECs treated with HEK293-exosomes containing CFZF-VPR relative to control HEK293 exosomes (HEK-Neg).

**
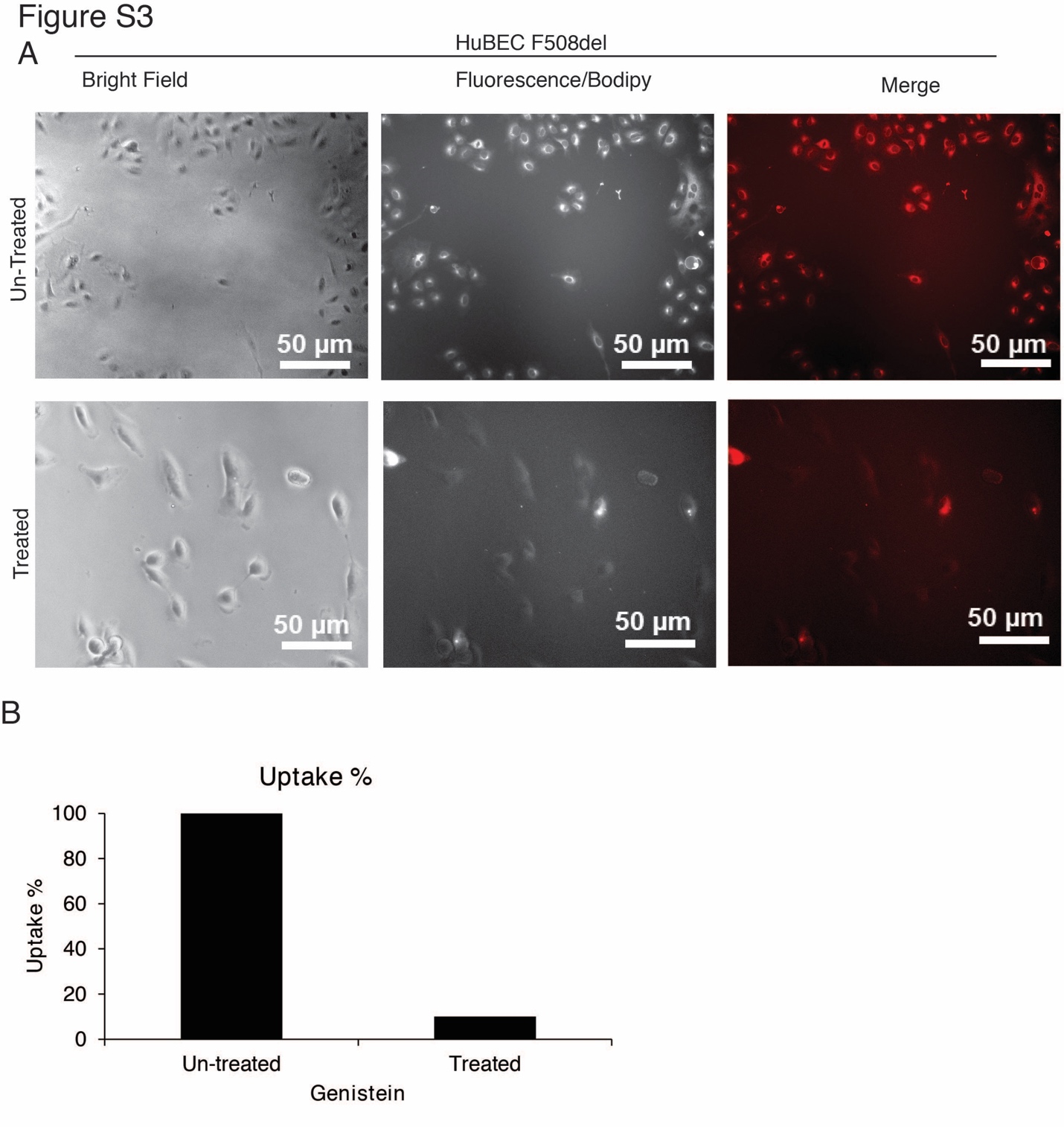
**

**Figure S3.** Exosome Uptake Inhibition. To study the pathway of exosome uptake, (**A**) HuBEC cells were pre-treated with 200 µM of Genistein and incubated at 37°C for 30 minutes before exosome addition, and then incubated with BODIPY-labeled exosomes at 37°C for 3h. (**B**) Uptake was quantified by determining the percentage of BODIPY- fluorescence intensity.

**Table S1.** Primer and CFZF DNA and protein sequences.

| **Name** | **Sequence (5’-3’)** |
| --- | --- |
| CFTR F | CTCATGGGATGTGATTCTTTCGACC |
| CFTR R | TTTCTGTCCAGGAGACAGGAGCAT |
| Beta Actin F | CACCAACTGGGACGACAT |
| Beta Actin R | ACAGCCTGGATAGCAACG |
| ZFA S1F | GGACTATAAGGACGACGATGACAAGA |
| ZFA S1R | GTATGGGTCCTCTGATGGCG |
| ZFA S2F | TGTCCCGAATGCGGCA |
| ZFA S2R | AGGGCGTCACTGCCG |
| CFTR Promoter F | CTGGGTCTGGCGGACCCTGA |
| CFTR Promoter R | TCGCGCGCGCTCCTTCCAGG |
| ZFP target CFTR P | GGGATGGGCCTGGTGCTGGGC |
| CFZF (zinc finer protein amino acid sequence) | LEPGEKPYKCPECGKSFSDPGHLVRHQRTHTGEKPYKCPECGKSFSRNDALTEHQRTHTGEKPYKCPECGKSFSRSDELVRHQRTHTGEKPYKCPECGKSFSRNDALTEHQRTHTGEKPYKCPECGKSFSDPGHLVRHQRTHTGEKPYKCPECGKSFSRRDELNVHQRTHTGEKPYKCPECGKSFSRSDKLVRHQRTHTGKKTS |
| VPR (protein amino acid sequence) | MLGSDALDDFDLDMLGSDALDDFDLDMLGSDALDDFDLDMLINSRSSGSPKKKRKVGSQYLPDTDDRHRIEEKRKRTYETFKSIMKKSPFSGPTDPRPPPRRIAVPSRSSASVPKPAPQPYPFTSSLSTINYDEFPTMVFPSGQISQASALAPAPPQVLPQAPAPAPAPAMVSALAQAPAPVPVLAPGPPQAVAPPAPKPTQAGEGTLSEALLQLQFDDEDLGALLGNSTDPAVFTDLASVDNSEFQQLLNQGIPVAPHTTEPMLMEYPEAITRLVTGAQRPPDPAPAPLGAPGLPNGLLSGDEDFSSIADMDFSALLGSGSGSRDSREGMFLPKPEAGSAISDVFEGREVCQPKRIRPFHPPGSPWANRPLPASLAPTPTGPVHEPVGSLTPAPVPQPLDPAPAVTPEASHLLEDPDEETSQAVKALREMADTVIPQKEEAAICGQMDLSHPPPRGHLDELTTTLESMTEDLNLDSPLTPELNEILDTFLNDECLLHAMHISTGLSIFDTSLF |
| CFZF+VPR DNA sequence | ATGGACTATAAGGACGACGATGACAAGAAGAGGCCAGCGGCTACTAAAAAGGCTGGACAGGCCAAaAAGAAaAAACTGGAGCCCGGCGAAAAGCCGTATAAGTGCCCAGAATGTGGGAAGAGTTTTTCTAGTAAGAAAGCATTGACGGAGCACCAGAGAACACACACAGGTGAAAAGCCTTACAAATGCCCTGAATGCGGTAAAAGCTTCAGTCGAGCGGATAATCTCACGGAGCATCAACGGACACACACTGGTGAAAAACCTTATAAATGTCCCGAATGCGGCAAATCTTTCAGTAGAAGTGATCATCTTACTAACCACCAGCGCACCCACACAGGCGAGAAACCGTATAAATGCCCCGAGTGTGGTAAGTCCTTTTCCGATAAAAAAGATCTTACTCGCCACCAACGCACCCACACGGGAGAAAAACCATATAAGTGTCCTGAGTGTGGCAAATCTTTTTCTAGGAGTGATAAACTTACGGAACACCAGAGGACTCATACTGGAGAGAAGCCATATAAATGTCCGGAATGCGGGAAAAGTTTTTCAGATCCAGGTCACTTGGTGCGCCATCAGAGGACCCATACGGGAAAAAAGACGAGCagcGCTGCTGACCCCAAGAAGAAGAGGAAGGTGTCGCCAGGGATCCGTCGACTTGACGCGTTGATATCAACAAGTTTGTACAAAAAAGCAGGCTACAAAGAGGCCAGCGGTTCCGGACGGGCTGACGCATTGGACGATTTTGATCTGGATATGCTGGGAAGTGACGCCCTCGATGATTTTGACCTTGACATGCTTGGTTCGGATGCCCTTGATGACTTTGACCTCGACATGCTCGGCAGTGACGCCCTTGATGATTTCGACCTGGACATGCTGATTAACTCTAGAAGTTCCGGATCTCCGAAAAAGAAACGCAAAGTTGGTAGCCAGTACCTGCCCGACACCGACGACCGGCACCGGATCGAGGAAAAGCGGAAGCGGACCTACGAGACATTCAAGAGCATCATGAAGAAGTCCCCCTTCAGCGGCCCCACCGACCCTAGACCTCCACCTAGAAGAATCGCCGTGCCCAGCAGATCCAGCGCCAGCGTGCCAAAACCTGCCCCCCAGCCTTACCCCTTCACCAGCAGCCTGAGCACCATCAACTACGACGAGTTCCCTACCATGGTGTTCCCCAGCGGCCAGATCTCTCAGGCCTCTGCTCTGGCTCCAGCCCCTCCTCAGGTGCTGCCTCAGGCTCCTGCTCCTGCACCAGCTCCAGCCATGGTGTCTGCACTGGCTCAGGCACCAGCACCCGTGCCTGTGCTGGCTCCTGGACCTCCACAGGCTGTGGCTCCACCAGCCCCTAAACCTACACAGGCCGGCGAGGGCACACTGTCTGAAGCTCTGCTGCAGCTGCAGTTCGACGACGAGGATCTGGGAGCCCTGCTGGGAAACAGCACCGATCCTGCCGTGTTCACCGACCTGGCCAGCGTGGACAACAGCGAGTTCCAGCAGCTGCTGAACCAGGGCATCCCTGTGGCCCCTCACACCACCGAGCCCATGCTGATGGAATACCCCGAGGCCATCACCCGGCTCGTGACAGGCGCTCAGAGGCCTCCTGATCCAGCTCCTGCCCCTCTGGGAGCACCAGGCCTGCCTAATGGACTGCTGTCTGGCGACGAGGACTTCAGCTCTATCGCCGATATGGATTTCTCAGCCTTGCTGGGCTCTGGCAGCGGCAGCCGGGATTCCAGGGAAGGGATGTTTTTGCCGAAGCCTGAGGCCGGCTCCGCTATTAGTGACGTGTTTGAGGGCCGCGAGGTGTGCCAGCCAAAACGAATCCGGCCATTTCATCCTCCAGGAAGTCCATGGGCCAACCGCCCACTCCCCGCCAGCCTCGCACCAACACCAACCGGTCCAGTACATGAGCCAGTCGGGTCACTGACCCCGGCACCAGTCCCTCAGCCACTGGATCCAGCGCCCGCAGTGACTCCCGAGGCCAGTCACCTGTTGGAGGATCCCGATGAAGAGACGAGCCAGGCTGTCAAAGCCCTTCGGGAGATGGCCGATACTGTGATTCCCCAGAAGGAAGAGGCTGCAATCTGTGGCCAAATGGACCTTTCCCATCCGCCCCCAAGGGGCCATCTGGATGAGCTGACAACCACACTTGAGTCCATGACCGAGGATCTGAACCTGGACTCACCCCTGACCCCGGAATTGAACGAGATTCTGGATACCTTCCTGAACGACGAGTGCCTCTTGCATGCCATGCATATCAGCACAGGACTGTCCATCTTCGACACATCTCTGTTTTGA |
